# Validation of the Ukrainian version of the Parental Burnout Assessment

**DOI:** 10.3389/fpsyg.2022.1059937

**Published:** 2022-12-05

**Authors:** Iryna Zbrodska, Isabelle Roskam, Lubov Dolynska, Moïra Mikolajczak

**Affiliations:** ^1^Department of Psychology, National Pedagogical Dragomanov University, Kyiv, Ukraine; ^2^Psychological Sciences Research Institute, UCLouvain, Louvain-la-Neuve, Belgium

**Keywords:** psychometric, validity, reliability, factor, parenting, burnout, Ukraine

## Abstract

The aim of the present study was to develop a Ukrainian version of the Parental Burnout Assessment (PBA) and examine its psychometric properties among Ukrainian parents. We examined the factorial structure of the Ukrainian version of the PBA (PBA-UA) and its relation with other variables, both antecedents and consequences of PB, in a sample of 1896 parents including 1735 (91,5%) mothers and 161 (8,5%) fathers. The original four-factor model (exhaustion from parental role, emotional distancing from one’s child, feelings of being fed up with parenting and contrast with previous parental self) and second-order model with a global parental burnout as a second-order factor fit the data well. The results of both subscale and global scores were reliable. The PBA-UA showed a positive association with perfectionism and general stress and a negative association with resilience. The low association with socio-demographic factors (i.e., marital status, number of children, number of children living in the household, work status) was replicated in accordance with previous studies of parental burnout, nevertheless mothers had higher level of parental burnout than fathers. PBA-UA also predicted both parental neglect and parental violence, even beyond general stress. The good psychometric properties of the PBA-UA suggests that this questionnaire can be used to assess parental burnout among Ukrainian parents.

## Introduction

The study of the phenomenon of parental burnout (PB) recently became an important issue in the field of mental health across the globe (Roskam et al., 2021). Every day parents face challenges of various intensity, going from providing for the basic needs for their children to more global issues related of caring for the children’s physical and mental health, and other concerns (education, development of their potential, …) which will affect their future. These demands place a heavy burden on parents’ shoulders and, in some circumstances, bring a high level of prolonged and overwhelming stress into their lives that can deplete a parent’s resources and lead to PB (Mikolajczak et al., 2018a,^2019^).

Thus, PB arises as a result from a chronic imbalance between risks over resources in the parenting domain (Mikolajczak and Roskam, 2018). It includes four dimensions: overwhelming exhaustion from parental role, emotional distancing from their children, feelings of being fed up in the parenting role or lack of pleasure in parenting, and contrast with previous parental self (Roskam et al., 2018). PB can have drastic consequences on a personal level (e.g., parental escapism, suicidal ideations, addictions, health disorders; Mikolajczak et al., 2018a,^2019^) and an interpersonal level (e.g., couple conflicts, violent and neglectful behavior toward children; Hubert and Aujoulat, 2018; Kawamoto et al., 2018; Mikolajczak and Roskam, 2018; Mikolajczak et al., 2019). Ultimately, the relationship between parents and children suffers the most. Studies show that the level of violence and neglect toward children increases significantly in parents who experience PB (Mikolajczak et al., 2019; Hansotte et al., 2021). This is concerning given that negative parenting behaviors, such as parental hostility toward children, can negatively affect children in both the short and long term and could be associated with child aggression and child behavior problems (Stormshak et al., 2000; Rubin and Burgess, 2002; Roelofs et al., 2006; Carrasco et al., 2009). Indeed, a study shows that mothers’ PB was related to adolescents’ perception of their mothers’ hostility and played a role in adolescents’ internalizing and externalizing problems (Chen et al., 2022).

## Parenting stress in Ukraine

The phenomenon of PB, its antecedents, risk factors, symptoms and consequences are actively studied in the world (see, e.g., Roskam et al., 2021, for a study involving 42 countries). Nevertheless, the subject of PB has not yet been scientifically explored in Ukraine. However, in the last 10 years, issues related to stress, exhaustion, high demands on parenting, especially motherhood, are increasingly discussed in pop science magazines, media, and internet communities, which demonstrates a strong interest in this phenomenon. That parents’ stress is a growing concern in Ukraine is not surprising: Since the beginning of the 21*^st^* century, Ukrainians have lived through two revolutions (2004, 2013-2014), a financial crisis (2007), the Crimean Peninsula annexation (2014) and, recently, war. The ongoing military conflict in the south-east part of Ukraine, started in 2014 and escalated into full-scale Russian aggression against Ukraine beginning in February 2022. Ukrainians live in political, economic, and social instability that bring a lot of distress, emotional tension, anxiety, disappointment, and emotional and physical fatigue.

Ukrainian parents face stress at the macro-, meso- and micro-levels. The strongest stress factors for parents on the macro level are fourfold: (1) war in the East of Ukraine since 2014 and ongoing tensions for the years since; (2) political instability (external and internal political situations); (3) increasing fear and uncertainty about the future of the country; and (4) economic instability (increased prices for basic utilities, food, and household necessities) leading to a higher cost of living. All these factors make parenting more complicated because they constantly worry about providing safety for their children and meeting their needs. Ensuring a decent future for the child is also becoming more difficult. At the meso-level, the lack of social support stemming from war-related relocations or even emigration is also a significant stress factor for parents. In addition to the above-mentioned factors that are more specific to the Ukrainian context, Ukrainian parents share with other parents in the world many micro-level risk factors such as parenting perfectionism, low self-esteem and feeling of incompetence in parenting, or child busy schedule.

Over the last two years, new stress factors further increased parenting stress. There was first the COVID-19 pandemic, including the lockdown of parents with their children, new conditions for the family’s functioning, fear that a child or partner will get sick, and distrust of the medical system. The last tremendous stressor for Ukrainian parents appeared on the 24*^th^* of February with the full-scale Russian invasion, which brought a completely new level of stress and uncertainty to the life of Ukrainian society, and parents in particular. With the beginning of the war, existing stressors intensified and new ones appeared, such as separation from a spouse or part of the family, leading to changes in family responsibilities and taking on new roles, lack of basic safety, a constant feeling of threat accompanied by anxiety; uncertainty and insecurity about the future; inability to plan, and general lack of stability; and concerns about the child’s psychological and general well-being in the future. War is a crisis on a social and intrafamily level that takes a huge portion of the parents’ energy, bringing additional risk factors to the development of children. Protecting one’s children and deciding on the best options to keep them safe, and striving to ensure their future became a challenge for many parents.

Although parental burnout has not yet been investigated in Ukraine, the foregoing stress factors make PB a likely phenomenon in this country. Disposing of a diagnostic instrument for PB among Ukrainian parents is urgent. It would not only allow researchers and mental health professionals to quantify the prevalence of PB in Ukraine, but also then to identify its level on early stages in order to help prevent it. The most widely used diagnostic tool for PBs is the Parental Burnout Assessment (PBA) (Roskam et al., 2018). This assessment has been translated and validated in many countries and successfully used all over the world and in countries as diverse as Finland (Aunola et al., 2020), Poland (Szczygieł et al., 2020), Romania (Stănculescu et al., 2020), Portugal (Matias et al., 2020), Turkey (Arikan et al., 2020) and China (Kawamoto et al., 2018) amongst several others. Nonetheless, these efforts have not yet been applied in Ukraine. Relying on the strong psychometric characteristics of the PBA demonstrated in the above research, this instrument is a good candidate for assessing PB in Ukraine.

## Purpose of the present study

The main goal of the present study is to examine the construct and concurrent validity of the Ukrainian version of the PBA (PBA-UA, see Supplementary Appendix A for the 23 items) among mothers and fathers living in Ukraine. Based on previous research, the items of the PBA-UA are expected to form four factors (first-order factor model): Emotional Exhaustion (EX), Emotional Distancing (ED), Feelings of Being Fed Up or Saturation (SA) and Contrast with Previous Parental Self (CO). In line with previous studies, we hypothesize these four factors are expected to load on a second-order factor “Parental Burnout” (Arikan et al., 2020; Aunola et al., 2020; Furutani et al., 2020; Gannagé et al., 2020; Matias et al., 2020; Mousavi et al., 2020; Stănculescu et al., 2020). It is expected that the reliabilities of the global score of the PBA-UA and its four subscales will all be satisfactory. The concurrent validity will be tested by examining the association of PB with five external variables: perfectionism, resilience, parental neglect, parental violence, and perceived stress. As detailed below, perfectionism and resilience were chosen to represent theoretical antecedents of PB. Neglect and violence were chosen to represent consequences of PB. Perceived stress is used both as a correlate and to examine the incremental validity of PB over general stress.

Perfectionism, the tendency to have very high standards for performance or behavior and be concerned to make mistakes (Frost et al., 1993; Slaney et al., 2001; Hill and Curran, 2016), has been found to be a strong risk factor for PB in several countries. A moderate aggregated correlation was found between parental perfectionism and PB in Belgian (r = 0.26) and Polish parents (r = 0.27) (Lin et al., 2021). Another study also showed a moderate correlation between parental perfectionism and PB (r = 0.32) in Polish parents (Lin and Szczygieł, 2022). A moderate correlation between general perfectionism and PB (r = 0.3) was found in Romanian parents (Stănculescu et al., 2020). A moderate to large correlation between parental perfectionism and PB (*r* = 0.34) was found in Japanese parents (Kawamoto et al., 2018), and a large correlation between maladaptive perfectionism and PB (r = 0.49) was found in Polish parents (Szczygieł et al., 2020). Based on these findings, a moderate positive correlation between PB and perfectionism was expected in the current study.

Resilience, the ability to resist adverse factors and to return to homeostasis after being exposed to stressors (Tugade et al., 2004; Smith et al., 2010), has also been found to be a strong protective factor against PB. Romanian researchers found a moderate negative association between resilience and PB (*r* =−0.36; Stănculescu et al., 2020) and Finnish researchers found a large negative association between resilience and PB (path coefficient =−0.47; Sorkkila and Aunola, 2022). Based on these studies, a moderate to large negative relationship between PB and resilience was expected in the present study.

Parental neglect and violence have been shown to be important and specific consequences of PB in cross-sectional, cross-lagged and experimental studies alike (Mikolajczak et al., 2018a,^2019^; Brianda et al., 2020). Belgian researchers found large positive correlations between PB and both neglect (r = 0.55) and violence (r = 0.51) (Mikolajczak et al., 2018a). Polish researchers found large positive correlations between PB and both neglect (r = 0.53) and violence (r = 0.42) (Szczygieł et al., 2020). Malaysian researchers also found large positive correlation between PB and both neglect (r = 0.44) and violence (r = 0.53) (Manja et al., 2020). Finnish researchers replicated these findings and found large positive correlation between PB and both neglect (*r* = 0.58) and violence (*r* = 0.54) (Aunola et al., 2021). Based on these studies, we also expect large positive correlations between PBA-UA and these two variables.

General perceived stress, the degree to which one’s life is appraised as stressful (Cohen et al., 1983), was measured here both as a correlate of PB but also, and more particularly, to test the incremental validity of PB to predict violence and neglect. In line with previous studies showing that PB predicts neglect and violence more than job burnout and depressive symptoms (Mikolajczak et al., 2020; Szczygieł et al., 2020), we hypothesize that PB will predict neglect and violence over and above general perceived stress.

Sociodemographic variables (i.e., parent’s gender, number of children, number of children living in the household, marital status, working status) were also measured but, based on previous studies (Gérain and Zech, 2018; Mikolajczak et al., 2018b; Sánchez-Rodrìguez et al., 2019; Stănculescu et al., 2020; Szczygieł et al., 2020), associations with PB are expected to be low. Nonetheless, based on previous recent findings, we hypothesize higher PB among mothers in comparison to fathers (Roskam and Mikolajczak, 2020; Szczygieł et al., 2020).

## Materials and methods

### Sample

The participants were 1896 parents including 1735 (91.5%) mothers and 161 (8.5%) fathers. Among the participants 99,84% had at least one child living with them permanently in the same household; three fathers (0.16%) have their children part-time at home. The survey was conducted in Ukrainian language. Most of the participants (99%) lived in Ukraine, 19 people (1.00%) currently lived abroad but identified as Ukrainian. Regarding age, 2.2% parents were aged 18 to 24 years old, 40.3% 25 to 34 years old, 46.4% 35 to 44 years old, 10.2% 45 to 54 years old, and 0.8% were older than 55 years old. Additionally, three people (0.16%) did not answer the question about age. Regarding marital status, 85.57% of parents lived with their partner, 14.06% lived separately, and seven respondents (0.37%) did not answer the question. The majority of parents had one (42.86%) or two (40.84%) children, 16.29% had tree and more children. Regarding the number of children actually living in the home, the number of families with one child was 47.50%, with two children was 38.87%, and three and more children was 13.63%.

Participants provided the age of their youngest child: 7.8% had a youngest child under one year old, 12.2% one to two years old, 12.9% between two and three years old, 17.7% between three and five years old, 12.5% between five and seven years old, 20.3% between seven and eleven years old, 9.5% between 11 and 14 years old, 5.6% between 14 and 18 years old, and 1.5% had a youngest child older than 18 years old.

Regarding employment status, 65.07% of the parents had at least a part-time job, while 34.93% had no job. The majority of participants (57.7%) were living in cities, 24.9% in the capital Kyiv, 6.8% in small towns and 10.5% in villages.

### Procedure

#### Parental Burnout Assessment translation procedure

The 23 items of the original version of the Parental Burnout Assessment (PBA, Roskam et al., 2018) were translated from English into Ukrainian by two professional translators and two Ukrainian researchers, including one of the authors. The resulting versions were discussed until agreement on a common version was obtained. The final version was back-translated into English with the help of another professional translator. This English version was presented to a native English speaker to judge the semantic similarity with the original version of the PBA. It was deemed very good. The face validity of the Ukrainian version of the PBA was confirmed by a focus group of 10 Ukrainian parents. Minor changes were made to further improve readability (Zbrodska, 2021).

#### Recruitment procedure

The data were collected from the beginning of November 2021 to the end of January 2022, just before Russia launched a full-scale invasion of Ukraine. Participants were recruited via social networks like Facebook or Instagram (where the study was advertised in groups for parents or on influencers’ pages), and via email invitation to friends, acquaintances, and participants of previous pilot studies on parental stress and exhaustion. It was also displayed on the Telegram channel “Psychological support”. The survey was presented online and information about the study was provided at the beginning of the survey. Parents were eligible to participate in the study only if they had at least one child still living at home. The responders participated voluntarily; no remuneration was offered. Also, the participants were assured that the data would remain anonymous and would be used for research purposes only. The respondents could answer the survey at a convenient time for them by filling in the online form.

### Measures

#### Parental burnout

Parental burnout was measured using the Ukrainian translation of the Parental Burnout Assessment described above. The original PBA questionnaire consists of 23 items forming four subscales: Emotional Exhaustion (nine items; e.g., “I feel completely run down by my role as a parent”), Emotional Distancing [three items; e.g., “I do what I’m supposed to do for my child(ren), but nothing more”], Saturation or Feelings of Being Fed Up with parenting (five items; e.g., “I feel like I can’t take any more as a parent”), and Contrast with Previous Parental Self (six items; e.g., “I tell myself that I’m no longer the parent I used to be”). Items are rated on a seven-point Likert scale from 0 (never), 1 (a few times a year or less), 2 (once a month or less), 3 (a few times a month), 4 (once a week), 5 (a few times a week), to 6 (and every day). Items are summed, such that the higher the score, the higher the level of PB. In the current study, Cronbach’s alpha was 0.94, 0.78, 0.91, 0.92 for the four subscales, respectively, and 0.97 for the global score.

#### Perfectionism

Perfectionism was measured via the Short Almost Perfect Scale (SAPS, Rice et al., 2014). The scale consists of eight items equally divided into two subscales: standards or high performance expectations (e.g., “I set very high standards for myself”) and discrepancy or, in other words, self-critical performance evaluation (e.g., “Doing my best never seems to be enough”). Items are rated on a 7-point Likert scale from 1 (strongly disagree) 2 (disagree) 3 (slightly disagree), 4 (neither), 5 (slightly agree), 6 (agree), to 7 (strongly agree). Subscale scores are calculated by averaging the responses to the items associated with each dimension. Higher scores reflect higher perfectionism. In the current study, Cronbach’s alpha was 0.85 for standards, 0.83 for discrepancy and 0.88 for the whole scale.

#### Parental neglect

Parental neglect was assessed using the short form of the Parental Neglect Scale (Mikolajczak et al., 2019), a 3-item questionnaire measuring emotional, educational and physical neglect [e.g., I don’t care about my children when I know I should (meals, hygiene, etc.)]. The questionnaire was translated into Ukrainian with the help of a professional translator. Also, adaptation, back translation and semantic analysis were accomplished. Items were rated on an 8-point scale from 1 (never), 2 (less than once a month), 3 (about once a month), 4 (a few times a month), 5 (about once a week), 6 (a few times a week), 7 (about once a day), to 8 (a few times a day). A global score was obtained by averaging the item scores. In the current study, Cronbach’s alpha was 0.69.

#### Parental violence

Parental violence was assessed with the short form of Parental Violence Scale (Mikolajczak et al., 2019), a 3-item questionnaire measuring psychological, physical and verbal violence toward a child [e.g., I say things to my children that I then regret (threats, insults, ridiculous nicknames, etc.)]. The questionnaire was translated into Ukrainian with the help of a professional translator. Also, adaptation, back translation and semantic analysis were accomplished. Items were rated on the same 8-point scale as for the Parental Neglect Scale. A global score was obtained by averaging the item scores. In the current study, Cronbach’s alpha was 0.71.

#### General stress

General stress was assessed using the Perceived Stress Scale (PSS; Cohen et al., 1983), which is a widely used stress assessment instrument. The tool also went through the translation/adaptation/back translation procedure. The PSS is 10-item questionnaire measuring the degree to which different situations are subjectively appraised as stressful by the individual. The questions ask about feelings and thoughts during the last month (e.g., “In the last month, how often have you felt nervous and stressed?”). The items were rated on a 5-point Likert-type scale: 0 (never), 1 (almost never), 2 (sometimes), 3 (fairly often), and 4 (very often). Individual scores are summed, with higher scores indicating higher perceived stress. In the current study, Cronbach’s alpha was 0.87.

### Data analysis procedure

We used Jamovi (Version 2.0.0.0) (for preliminary and descriptive and hierarchical linear regression analyses) and JASP (Version 0.14.1 for factor analyses) software to analyze the data. The first step was to exclude the data that did not fit the criteria of the research. Since the present study explores PB among Ukrainian parents who live in Ukraine and since living in another country, a parent could be exposed to stress factors different from those that are prevalent in Ukraine, which can affect the level of PB differently, 20 records were excluded: 19 of the respondents do not live in Ukraine and there was one duplicate record.

As responders participated in the study on a volunteer basis, in preparing the study we tried to make it as easy as possible for them to fill out the questionnaire. Because of this, participants’ ages were split into the age ranges as presented in the article, rather than having them type their age. Looking back, we see that this was not the best decision, but, unfortunately, we cannot change the design in retrospect.

Descriptive statistics had been conducted to obtain the descriptive information (such as minimum and maximum scores, mean, standard deviation, kurtosis, and skewness) for all variables. We tested the normality of the data distribution through the evaluation of skewness and kurtosis indicators. The values for asymmetry between -2 and +2 for skewness and between -7 and +7 for kurtosis were considered acceptable (Byrne, 2010; Hair et al., 2010). No data transformation was applied and Pearson correlations were used. To examine the factor structure of the Ukrainian version of the PBA we used confirmatory factor analysis, using the diagonally weighted least squares (DWLS) estimation method (Brown, 2015). We first tested a first-order factor model where the 23 items of the PBA form four correlated factors (like in Roskam et al., 2018). We then tested a second-order factor model in which the 23 items of the PBA form four first-order factors that all load on a second-order factor (like in, e.g., Arikan et al., 2020; Aunola et al., 2020; Furutani et al., 2020; Gannagé et al., 2020; Matias et al., 2020; Mousavi et al., 2020; Stănculescu et al., 2020). The fit indices used to evaluate the adequacy of the models were: Root Mean Square Error of Approximation (RMSEA), Standardized Root Mean Square Residual (SRMR), Comparative Fit Index (CFI) and Tucker-Lewis Index (TLI). The following reference values were considered: CFI and TLI values > 0.90 and preferably above 0.95; RMSEA values < 0.08 or preferably less than 0.06 (with the upper limit of the confidence interval < 0.10); SRMR values < 0.08. Standardized factor loadings greater than 0.40 were also considered adequate (Hu and Bentler, 1999; Brown, 2015). The reliability of the questionnaires was examined using Cronbach’s Alphas.

Pearson’s r correlation analyses were conducted to test concurrent validity and the relationship between PBA (and its four dimensions) and perfectionism, resilience, perceived stress, parental neglect, and parental violence. Regression analysis was conducted to determine the relative weight of the four dimensions of the PBA. Finally, hierarchical linear regression analysis was used to test the hypothesis that PB has incremental validity over general perceived stress to predict neglect and violence.

## Results

### Confirmatory factor analysis

**FIGURE 1:**
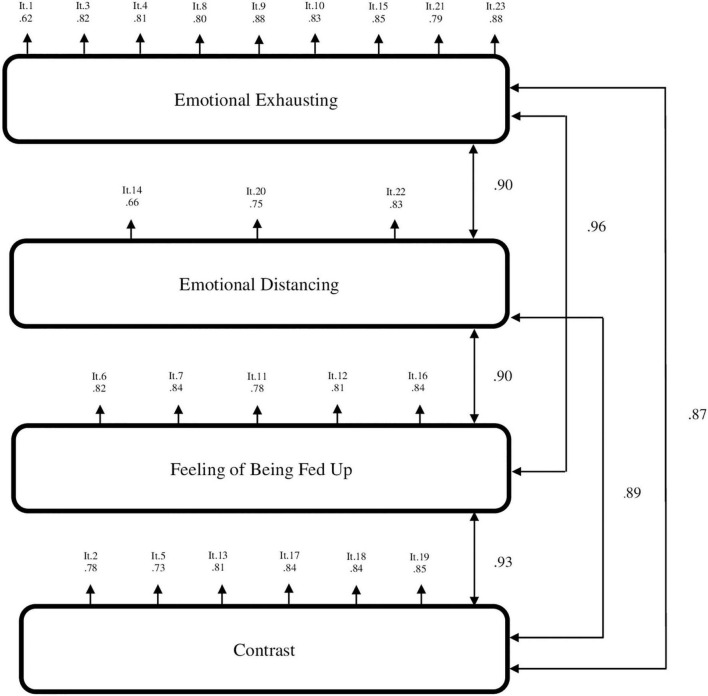
Results of the confirmatory factor analysis for the first-order factor model of the PBA-UA (*N* = 1878). Emotional Exhausting – emotional exhausting from parental role; Emotional Distancing – emotional distancing from one’s child; Feeling of Being Fed Up – feeling of being fed up with parenting; Contrast – contrast with previous parental self; It – item number in the order in which it is presented in the assessment.

**FIGURE 2:**
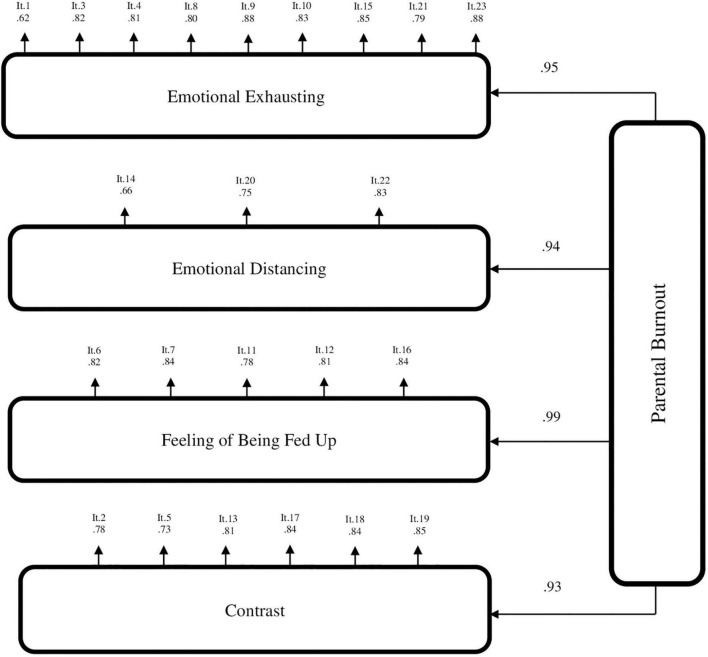
Results of the confirmatory factor analysis for the second-order factor model of the PBA-UA (*N* = 1878). Emotional Exhausting – emotional exhausting from parental role; Emotional Distancing – emotional distancing from one’s child; Feeling of Being Fed Up – feeling of being fed up with parenting; Contrast – contrast with previous parental self; It – item number in the order in which it is presented in the assessment.

First, we tested the first-order factor model. The results showed a good fit to the data with CFI = 0.99, TLI = 0.99, RMSEA = 0.03, CI 90% (0.031,0.037). S-Bχ2(224) = 707.25 was significant at *p* < 0.001, which may indicate some discrepancy between the hypothetical model and the data, but χ2 tends to increase in large samples. All the estimated factor loadings found in the CFA for the first-order factor model were significant at *p* < 0.001. Standardized factor loadings ranged between.63 and.88 (see Figure 1 for factor loadings). Regarding the second-order factor model with the four factors as first-order factors and “Parental burnout” as the second-order factor, the model showed a good fit to the data: with CFI = 0.99, TLI = 0.99, RMSEA = 0.03 CI 90% (0.032,0.037). S-Bχ2(226) = 726.43 was significant at *p* < 0.001, but χ2 tends to increase in large samples. All the estimated factor loadings found in the CFA for the second-order factor model were significant at *p* < 0.001. Standardized factor loadings ranged between 0.62 and 0.88 (see Figure 2 for factor loadings). The chi-square difference test [χ2diff (2) = 19.177, *p* < 0.001] showed that there is some discrepancy between the first-order factor model and the second-order factor model. However, according to other fit indices (CFI, TLI, RMSEA, SRMR) the both models could be considered to fit the data equally well and supported the construct validity of the models.

### Reliability

Regarding the reliability for the PBA-UA, the results demonstrate high levels of internal consistency: Cronbach’s alpha for the global score of the PBA-UA was α = 0.97 (0.97 for mothers and 0.94 for fathers), for Exhaustion from parental role it was α = 0.94 (0.94 for mothers and 0.91 for fathers), for Emotional distancing from the one’s child it was α = 0.78 (0.79 for mothers and 0.70 for fathers), for Feeling of being fed up in the parenting role it was α = 0.91 (0.91 for mothers and 0.89 for fathers), and for Contrast with previous parental self, it was α = 0.92 (0.92 for mothers and 0.91 for fathers).

### Relationships with other variables

The concurrent validity was tested by examining the association of PBA-UA with the following external variables: perfectionism, resilience, parental neglect, parental violence, and perceived stress. The correlation between PBA-UA (both global score and subscale scores) and all the variables under study are displayed in Table 1. Specifically, the correlation between PBA-UA global score and perfectionism was 0.28 (0.05 for standards subscale, but 0.35 for discrepancy subscale), −0.31 for resilience, 0.33 for general stress, 0.45 for parental neglect, and 0.50 for parental violence.

**TABLE 1:** Correlations between parental burnout, perfectionism, resilience, stress, parental neglect, and parental violence (*N* = 1878).

		1.	2.	3.	4.	5.	6.	7.	8.	9.	10.	11.	12.
1.	PBA-UA	–											
2.	PBA (EX)	**0.96**	–										
3.	PBA (ED)	**0.85**	**0.78**	–									
4.	PBA (FU)	**0.94**	**0.89**	**0.76**	–								
5.	PBA (CO)	**0.92**	**0.81**	**0.76**	**0.84**	–							
6.	SAPS	**0.28**	**0.21**	**0.17**	**0.20**	**0.24**	–						
7.	SAPS (ST)	0.05	0.05	0.01	0.05	0.06	**0.88**	–					
8.	SAPS (DI)	**0.35**	**0.33**	**0.29**	**0.31**	**0.37**	**0.87**	**0.53**	–				
9.	BRS	−**0.31**	−**0.30**	−**0.24**	−**0.28**	−**0.31**	−**0.22**	–0.03	−**0.35**	–			
10.	PSS	**0.33**	**0.33**	**0.27**	**0.31**	**0.31**	**0.29**	**0.20**	**0.31**	−**0.26**	–		
11.	PNS	**0.45**	**0.40**	**0.49**	**0.42**	**0.41**	**0.08**	–0.04	**0.19**	−**0.11**	**0**.**18**	–	
12.	PVS	**0.50**	**0.44**	**0.44**	**0.47**	**0.52**	**0.09**	–0.02	**0.18**	−**0.18**	**0**.**17**	**0**.**40**	–

Due to the number of correlations, we applied the Bonferroni correction. A correlation is deemed significant only if the p-value is equal or lower than 0.001. Significant correlations after Bonferroni correction are in bold. PBA-UA – Parental Burnout Assessment, global score (23 items), PBA (EX) – exhaustion from the parenting (9 items), PBA (ED) – emotional distancing from one’s child (3 items), PBA (FU) – feeling of being fed up with parenting (5 items), PBA (CO) – contrast with previous parental self (6 items), SAPS – Short Almost Perfect Scale (8 items), SAPS (ST) – Standards (4 items), SAPS (DI) – Discrepancy (4 items), BRS – Brief Resilience Scale (6 items), PSS – Perceived Stress Scale (10 items), PNS – Parental Neglect Scale (3 items), PVS – Parental Violence Scale (3 items).

The association between PB and socio-demographic variables (i.e., parent’s gender, marital status, number of children, number of children living in household and working status) is shown in Table 2. In line with our expectations, gender-related differences were found: Mothers had significantly higher PBA-UA scores than fathers. This was true for the global score, F(1,1876) = 51.46, p < 0.001, as well as for the four dimensions of the PBA-UA with, respectively: F(1, 1876) = 58.52, p < 0.001 for exhaustion from parental role, F(1, 1876) = 19.45, p < 0.001 for emotional distancing from one’s child, F(1, 1876) = 41.04, p < 0.001 for feelings of being fed up with parenting, and F (1, 1876) = 43.10, p < 0.001 for contrast with previous parental self. By contrast, there was no significant difference in PB levels between parents who live with or without a partner, F (1,1876) = 0.50, p = 0.48. There were also no significant differences in PB levels between parents with different numbers of children F (2, 1877) = 2.49, p = 0.083, but there was a difference in PB levels between parents with different numbers of children living in the household, F (2, 1877) = 7.14, p = 0.001. Bonferroni’s *post hoc* test showed that parents with one child had lower levels of PB than parents with two (ΔM =−4.37, p = 0.021) and three children (ΔM =−7.76, p = 0.002) living in the household, respectively. No differences were found between parents who had two and three children in the household (ΔM =−3.38, p = 0.46). Finally, non-working parents reported higher burnout levels than working parents, F (1,1876) = 31.16, p < 0.001.

**TABLE 2:** Descriptive statistics of the PBA-UA subscales and global score according to gender, marital status, number of children, number of children living in household and working status.

	Sample	Gender		Marital status		Number of children			Number of children living in the household			Work status	
						
	M (SD) (*N* = 1878) M (SD)	Mothers (*N* = 1,718) M (SD)	Fathers (*N* = 160) M (SD)	Living together (*N* = 1607) M (SD)	Living separately (*N* = 264) M (SD)	One (*N* = 805) M (SD)	Two (*N* = 767) M (SD)	Three and more (*N* = 306) M (SD)	One (*N* = 892) M (SD)	Two (*N* = 730) M (SD)	Three and more (*N* = 256) M (SD)	At least part time job (*N* = 1222) M (SD)	No job (*N* = 656) M (SD)
PBA (EX)	16.29 (13.89)	17.01 (13.99)	8.44 (9.50)	16.39 (13.59)	16.39 (15.29)	15.59 (13.69)	16.69 (13.79)	17.29 (14.19)	15.19 (13.69)	16.99 (13.79)	18.49 (14.19)	14.59 (13.29)	19.49 (14.39)
PBA (ED)	5.32 (4.41)	5.46 (4.46)	3.86 (3.54)	5.29 (4.36)	5.56 (4.69)	4.92 (4.21)	5.54 (4.48)	5.83 (4.67)	4.88 (4.29)	5.57 (4.39)	6.13 (4.68)	5.14 (4.32)	5.66 (4.55)
PBA (SA)	6.86 (7.19)	7.19 (7.29)	3.41 (4.87)	6.82 (7.08)	7.18 (7.95)	6.64 (7.14)	6.95 (7.21)	7.24 (7.33)	6.45 (7.18)	7.03 (7.11)	7.81 (7.47)	6.22 (6.97)	8.06 (7.47)
PBA (CO)	9.93 (9.45)	10.39 (9.55)	5.29 (6.86)	9.81 (9.33)	10.79 (10.19)	9.36 (9.26)	10.29 (9.49)	10.39 (9.79)	9.22 (9.24)	10.39 (9.45)	10.99 (9.99)	9.41 (9.29)	10.89 (9.68)
PBA – UA	38.39 (32.60)	40.09 (32.89)	20.99 (23.09)	38.28 (32.10)	39.98 (35.77)	36.59 (31.99)	39.49 (32.69)	40.69 (33.69)	35.69 (32.09)	40.09 (32.39)	43.39 (33.99)	35.39 (31.69)	44.09 (33.49)
Statistic for the global score		*F*(1,1876) = 51.46, *p* < 0.001 Cohen’s *d* = 0.59		*F*(1,1876) = 0.50, *p* = 0.48 Cohen’s *d* = 0.05		*F*(2, 1875) = 2.49, *p* = 0.083 Cohen’s *d* = 0.11			*F*(2, 1875) = 7.14, *p* < 0.001 Cohen’s *d* = 0.18			*F*(1,1876) = 31.16, *p* < 0.001 Cohen’s *d* = 0.27	

PBA-UA – Parental Burnout Assessment, global score, PBA (EX) – exhaustion from parenting, PBA (ED) – emotional distancing from one’s child, PBA (FU) – feeling of being fed up with parental role (5 items), PBA (CO) – contrast with previous parental self.

### Incremental validity

A study of the relationship between parental neglect and parental violence, and the two predictors of PB and general stress, fully confirmed our hypothesis that PB is a stronger predictor of parental neglect and violence than general stress. As shown in Tables 3, 4, general stress no more predicts neglect and violence in the presence of PB.

**TABLE 3:** Regression model predicting parental neglect.

Predictors	Stand. estimate	t	p	R^2^
PSS	0.0324	1.48	0.139	0.0319
PBA	0.4376	19.99	<0.001	0.2020

**TABLE 4:** Regression model predicting parental violence.

Predictors	Stand. estimate	t	p	R^2^
PSS	0.0095	0.446	0.655	0.0304
PBA	0.4937	23.221	<0.001	0.2470

### Prevalence of parental burnout and perceived general stress

Regarding the prevalence of PB, respondents were considered to experience PB if they scored 92 or higher on the PBA, which is the mean score of a parent who experiences every symptom at least once a week (Szczygieł et al., 2020; Sarrionandia and Aliri, 2021; Hamvai et al., 2022). Using this cut-off, the prevalence rate of burnout stood at 10.17% in the total sample; 10.83% for mothers and 3.13% for fathers.

Regarding the prevalence of general perceived stress, respondents were considered to have high perceived stress if the total score ranged from 27 to 40 (Cohen et al., 1983). Using this cut-off, the prevalence rate of perceived stress stood at 20.02% in the total sample. The mean score of the scare was 28.49 (SD 1.85). Regarding gender difference the prevalence rate of perceived stress for mothers was 20.61% with a mean of 28.53 (SD 1.87) and for fathers it was 13.75% with a mean of 28.00 (SD 1.57).

## Discussion

In this study, we assessed the psychometric properties of the Ukrainian version of the PBA. The original PBA is a valid and reliable method used in more than 40 countries for assessing PB (Roskam et al., 2018; Roskam et al., 2021). The results demonstrated strong psychometric properties within the PBA-UA, in terms of reliability and validity of its scores. Our findings are consistent with the results of the original version of the PBA (Roskam et al., 2018).

Regarding factor structure, our study replicated the first-order factor model previously found in the original version of the PBA (Roskam et al., 2018). However, and in line with previous studies, the second-order factor model, with the four factors as the first-order factor and “Parental burnout” as the second-order factor, also fit the data. Our findings therefore support the concept of PB. It allows us to use not only the subscale scores, but the total score of the PBA (Arikan et al., 2020; Aunola et al., 2020; Furutani et al., 2020; Gannagé et al., 2020; Matias et al., 2020; Mousavi et al., 2020; Stănculescu et al., 2020; Szczygieł et al., 2020).

Evidence for the validity of the PBA-UA also lies in the correlations of the PBA-UA with the variables considered to be antecedents and consequences of PB. Our results first replicate the positive relationship between perfectionism and PB found in previous studies. The association is stronger between PB and the discrepancy subscale, which confirms the findings of other researchers, namely, that intolerance for shortcomings and self-critical evaluation increase the risk of PB more than high parenting standards (Kawamoto et al., 2018; Stănculescu et al., 2020; Szczygieł et al., 2020; Lin et al., 2021; Lin and Szczygieł, 2022). This study also replicates the negative association between PB and resilience found in previous studies (Stănculescu et al., 2020; Sorkkila and Aunola, 2022), as well as the strong positive association between PB and parental neglect and violence (Mikolajczak et al., 2018a,^2019^; Aunola et al., 2020; Manja et al., 2020; Szczygieł et al., 2020; Brianda et al., 2020).

In the present study we presented a preliminary prevalence of PB in the Ukrainian population. The obtained data showed that the prevalence of PB was 10.17% of the total sample, regarding the gender difference it was 10.83% for mothers and 3.13% for fathers. Our results showed higher levels of PB in comparison to participants from 42 other countries. Previously, the highest preval1ence rates of PB were found in Belgium (8.1% for the total sample), Poland (7.7% for the total sample), Canada (6,5% for the total sample) (Roskam et al., 2021), and Hungary [5.8% of the total sample (Hamvai et al., 2022)]. Thus, PB, appears to be an urgent problem in Ukraine. Even before the full-scale invasion of Russia parents in Ukraine had the highest levels of PB among other European countries. This is a serious challenge for researchers and clinicians in Ukraine, especially in the context of long-term stress related to military actions and trauma.

It is important to note that, as expected, the effect of PB on parental neglect and violence toward one’s children is significantly higher than the effect of general stress. Our results support previous research, which shows that parental neglect and violence are more specific consequences of PB than that of general stress or distress (Mikolajczak et al., 2020; Szczygieł et al., 2020). This finding is important in Ukraine where the level of general stress is relatively high. Finally, the Ukrainian sample has a significantly higher mean score [28.49 (SD 1.85)] in comparison to other countries, where the mean score was 17.4 (SD 6.5) (Gamonal-Limcaoco et al., 2022).

We also replicate the previous findings that mothers have a higher level of PB than fathers (Furutani et al., 2020; Gannagé et al., 2020; Stănculescu et al., 2020; Szczygieł et al., 2020; Sarrionandia and Aliri, 2021), as well as parents who have more children living in the household (Roskam et al., 2018; Gannagé et al., 2020; Mousavi et al., 2020; Stănculescu et al., 2020; Szczygieł et al., 2020), and non-working parent (Arikan et al., 2020; Mousavi et al., 2020; Stănculescu et al., 2020; Szczygieł et al., 2020). It is to be added that the impact of sociodemographic factors on PB was rather low, as in previous studies (Mikolajczak and Roskam, 2018; Mikolajczak et al., 2018b; Stănculescu et al., 2020; Szczygieł et al., 2020). Possibly having a job gives parents a feeling of stability, financial security and self-realization, which can be a factor in decreasing PB.

In conclusion, this study both expands the growing evidence on PB and provides empirical support for the validity of the PBA-UA which can now be used for assessing PB in Ukraine. The availability of a validated tool will be most useful in the diagnosis and understanding of this phenomenon for specialists in the psychological and social fields who work with parents or families. It can also be used to assess the effectiveness of programs to prevent or treat PB. In light of this, the fact that the PBA is available through open access is an asset. Additionally, the availability of the PBA-UA will allow Ukrainian researchers to participate actively in both national and cross-cultural research aimed at better understanding the causes and consequences of PB.

### Limitations and directions for future research

While our study provided evidence for the validity of the PBA-UA, this study has several limitations. First, although the present study was carried out among a relatively large sample, the majority of participants were mothers. Fathers’ participation in parental burnout studies in other countries is also lower than that of mothers (Roskam et al., 2018; Aunola et al., 2020; Hamvai et al., 2022). However, in our sample, this difference is especially notable. It is known that in online data collection, the participation of women tends to be higher than that of men (Stoop, 2005; Stoop et al., 2010). While the reason for low participation from fathers is unknown, we hypothesize that it could have happened because the participants were not provided financial incentives for their participation. Also, in Ukrainian culture, raising children, including participating in activities related to childcare and upbringing (e.g., development, education) is seen mostly as the mother’ responsibility (Libanova et al., 2009). That is why it is also possible that parental burnout is seen as a more pressing issue for mothers, which made them more readily interested in participating in the study. Regardless, caution should be exercised when extrapolating the present results for fathers to the entire male population of Ukraine due to the small male sample size in the present study. Second, the data collection was based solely on self-reports. In addition to overcoming these shortcomings, future research in the Ukrainian context will need to dig deeper into the specific antecedents of PB in Ukraine at the macro-, meso- and micro-levels (e.g., the possible influence of war trauma on PB as well as for the specific consequences that PB may have in this country and context).

## Data availability statement

The raw data supporting the conclusions of this article will be made available by the authors, without undue reservation.

## Ethics statement

The research with human participants was reviewed and approved by the Scientific and Methodological Board of the Department of Psychology at National Pedagogical Dragomanov University, Kyiv, Ukraine. Written informed consent for participation was not required for this study in accordance with the national legislation and the institutional requirements.

## Author contributions

All authors listed have made a substantial, direct, and intellectual contribution to the work, and approved it for publication.
