# Supplementary material for: Validation of the Ukrainian version of the Parental Burnout Assessment
Source: Front Psychol. 2022 Dec 5;13:1059937. doi: 10.3389/fpsyg.2022.1059937 (PMC9760865; doi:10.3389/fpsyg.2022.1059937)
Supplement: Supplementary file 1 [file Data_Sheet_1.docx]

**Опитувальник батьківського вигорання**

Діти – важливе джерело задоволення та радості для батьків. Водночас для деяких батьків вони також можуть стати джерелом виснаження. Тут немає протиріччя, задоволення від батьківства та одночасно виснаження від батьківської ролі/обов'язків можуть співіснувати. Це можливо – любити своїх дітей, але в той же час відчувати виснаження або знесилення у своїй батьківській ролі.

Представлений нижче опитувальник, стосується виключно відчуття виснаження, яке можуть переживати батьки (як мами, так і тата). Для кожного твердження оберіть відповідь, яка найкраще відповідає тому, що відчуваєте особисто ви.

Тут немає правильних чи неправильних відповідей. Якщо у вас ніколи не було такого відчуття, виберіть «Ніколи». Якщо у вас бувало таке відчуття, виберіть одну відповідь на шкалі від "Декілька разів на рік" до "Щодня", яка якнайкраще описує, як часто ви себе так почували.

|  |  | **Ніколи** | **Декілька разів на рік** | **Один раз на місяць або менше** | **Декілька разів на місяць** | **Один раз на тиждень** | **Декілька разів на тиждень** | **Кожного дня** |
| --- | --- | --- | --- | --- | --- | --- | --- | --- |
| 1 | Я настільки знесилилась(вся) від батьківської ролі (обов’язків), що сон вже не приносить бажаного відпочинку. | 0 | 1 | 2 | 3 | 4 | 5 | 6 |
| 2 | Мені здається, що як мама/тато я дезорієнтована(ий) і втратила(в) напрямок у своєму материнстві/батьківстві. | 0 | 1 | 2 | 3 | 4 | 5 | 6 |
| 3 | Я почуваюся повністю змученою(им) від своїх батьківських обов’язків. | 0 | 1 | 2 | 3 | 4 | 5 | 6 |
| 4 | У мене взагалі немає сил, щоб піклуватися про свою дитину (дітей) | 0 | 1 | 2 | 3 | 4 | 5 | 6 |
| 5 | Я думаю, я не така(ий) хороша(ий) мама/тато, якою(им) була(в) раніше по відношенню до своєї дитини (дітей). | 0 | 1 | 2 | 3 | 4 | 5 | 6 |
| 6 | Батьківство стає для мене нестерпним. | 0 | 1 | 2 | 3 | 4 | 5 | 6 |
| 7 | Я відчуваю, що моє батьківство – надто тяжка ноша для мене і я знаходжуся «на межі». | 0 | 1 | 2 | 3 | 4 | 5 | 6 |
| 8 | У мене виникає відчуття, що я доглядаю за дитиною (дітьми) на автопілоті. | 0 | 1 | 2 | 3 | 4 | 5 | 6 |
| 9 | Мені здається, що я дуже знесилилася(вся) від батьківства. | 0 | 1 | 2 | 3 | 4 | 5 | 6 |
| 10 | Коли я прокидаюся вранці і попереду на мене чекає черговий день з моєю дитиною (дітьми), то я вже почуваюся виснаженою(им) навіть до того, як почну щось робити. | 0 | 1 | 2 | 3 | 4 | 5 | 6 |
| 11 | Час з дитиною (дітьми) не приносить мені задоволення. | 0 | 1 | 2 | 3 | 4 | 5 | 6 |
| 12 | Мені здається, що як мама/тато, я не справляюсь. | 0 | 1 | 2 | 3 | 4 | 5 | 6 |
| 13 | Я зізнаюсь собі, що я вже не та мама/той тато, якою(им) була(в) раніше. | 0 | 1 | 2 | 3 | 4 | 5 | 6 |
| 14 | Я роблю необхідний мінімум для дитини (дітей), але не більше. | 0 | 1 | 2 | 3 | 4 | 5 | 6 |
| 15 | Батьківські обов’язки висмоктують з мене всі ресурси. | 0 | 1 | 2 | 3 | 4 | 5 | 6 |
| 16 | Я більше не витримую бути мамою/татом. | 0 | 1 | 2 | 3 | 4 | 5 | 6 |
| 17 | Мені соромно за те, якою мамою/татом я стала(в). | 0 | 1 | 2 | 3 | 4 | 5 | 6 |
| 18 | Я більше не пишаюсь собою, як мамою/татом. | 0 | 1 | 2 | 3 | 4 | 5 | 6 |
| 19 | Коли я взаємодію з дитиною(дітьми), у мене складається враження, що я – це вже не я. | 0 | 1 | 2 | 3 | 4 | 5 | 6 |
| 20 | Я більше не здатна(ий) показувати своїй дитині (дітям) як сильно я її/їх люблю. | 0 | 1 | 2 | 3 | 4 | 5 | 6 |
| 21 | В мене опускаються руки навіть від думок про все те, що я маю зробити для своєї дитини (дітей). | 0 | 1 | 2 | 3 | 4 | 5 | 6 |
| 22 | Окрім звичайної рутини (відвезти/привезти, вкласти спати, приготувати їжу, нагодувати), в мене не вистачає енергії на додаткові зусилля для моєї дитини (дітей). | 0 | 1 | 2 | 3 | 4 | 5 | 6 |
| 23 | Я проживаю своє батьківство в «режимі виживання». | 0 | 1 | 2 | 3 | 4 | 5 | 6 |

**Якщо ваш результат 30 або менше балів:**

**У вас вигорання відсутнє**

На даний момент вам не загрожує батьківське вигорання. Це не означає, що у вас ніколи раніше не було вигорання, що у вас ніколи не буде ризику вигоріти або ви ніколи не будете мати досвіду вигорання. Набраний бал фактично означає, що в даний час ваші фактори захисту перевищують фактори ризику. Зараз у вас є енергія та ресурси взяти на себе батьківську роль та справлятись з будь-якими труднощами та проблемами, які можуть виникнути. Ви віддані вихованню ваших дітей та інвестуєте в побудову стосунків з ними. Загалом, ви щаслива(ий) мама/тато.

**Якщо ваш результат від 31 до 45 балів включно:**

**У вас низький ризик батьківського вигорання**

Наразі, ризик вигорання низький. В даний момент ваші фактори захисту перевершують фактори ризику. Навіть якщо ви час від часу відчуваєте втому, у вас вистачає енергії та ресурсів, щоб впоратися з повсякденним життям та проблемами. Ви вкладаєтесь у виховання та стосунки зі своїми дітьми. І хоча, часом, ви хочете зробити більше, загалом, ви почуваєтесь хорошою(им) мамою/татом. Для того, щоб зберегти та підтримувати цей баланс, обов’язково піклуйтеся про свої фактори захисту, наприклад, емоційні ресурси, якість подружніх стосунків та стосунків з дітьми.

**Якщо ваш результат від 46 до 60 балів включно:**

**У вас помірний ризик батьківського вигорання**

В даний час у вас помірний ризик вигорання. Це не означає, що ви вигорите, але означає, що пора почати піклуватися про себе. Звичайно, ви, в цілому, все ще в хорошій формі, ви все ще дбаєте про виховання своїх дітей, а стосунки, які ви маєте з ними, дуже важливі для вас. Ви все ще добре виконуєте свої батьківські/материнські обов’язки. Але іноді ви відчуваєте, що в якості матері/батька, вам необхідно надто багато з чим справлятись. Тоді відчувається втома, і іноді, це заважає вам залучатись у стосунки зі своїми дітьми на стільки, на скільки вам хотілося б. Іноді виникає відчуття, що ви не така(ий) мама/тато, яким б ви хотіли бути. Щоб уникнути ризику вигорання, важливо підсилити фактори захисту, що зменшують батьківський стрес та/або зменшити фактори, що його збільшують.

**Якщо ваш результат від 61 до 75 балів включно:**

**У вас високий ризик батьківського вигорання**

На даний момент у вас високий ризик батьківського вигорання. Можливо, ви вже однією ногою там. Виглядає так, що основною проблемою є втома та виснаження. Все частіше ви опиняєтесь «на межі», відчуваючи, що просто не можете більше цього робити. Ви намагаєтесь не здатися, намагаєтесь «триматись курсу» щодо своїх піклування про своїх дітей, але ваша здатність справлятися з батьківськими обов’язками «тріщить по швам». Можливо, це вже має певний вплив і на ваше здоров’я. Також можливо, що споживання кофеїну, тютюну або алкоголю збільшилось. Можливо, ви стали більш уразливі, більш дратівливі, і це впливає на стосунки з вашою половинкою та/або стосунки з дітьми: ви злитеся/дратуєтесь швидше, ніж зазвичай, терпіння теж закінчується швидше. Ви ухиляєтесь від деяких батьківських завдань. Ви помічаєте, що більше не є тим татом чи тією мамою, якими були раніше. Ви любите своїх дітей, але іноді вам просто хочеться відпочити від батьківської ролі. Вам хочеться знайти час для себе. На цьому етапі, надзвичайно важливо, щоб ви знайшли можливість і звільнили час особисто для себе, з одного боку, і спільний час з вашим партнером, з іншого.

**Якщо ваш результат від 76 до 84 балів включно:**

**Ймовірно, у вас батьківське вигорання**

Ви можете бути одним із батьків, які зараз мають батьківське вигорання. Ви регулярно знесилені, заморені, безсилі, виснажені. Іноді просто думка про те, що треба пережити новий день здається вам нестерпною. Ви все ще намагаєтесь триматися, але часом у вас залишається так мало енергії, що ви все частіше робите речі механічно: робота чи щоденні обов’язки - вечеря - сон. Щодо дітей - забезпечуєте найнеобхідніше, слухаєте у пів-вуха...з думкою, що необхідно протриматися ще один день. Є гостре бажання, щоб вони якнайшвидше були в ліжку. Але потім з’являється почуття провини, бо ви були недостатньо залучені або «не були присутні» по-справжньому або за те, що злились більше, ніж мали б. Ви усвідомлюєте, що ви вже не є тим татом або тією мамою, якими були раніше чи якими хотіли би бути. Ви бажаєте бути деінде. В той же час відчуваєте себе винними, бо ви погана мама чи поганий батько. Ви виснажуєте себе почуттям провини, але все одно намагаєтесь виконувати те, що необхідно. Часто ви настільки втомлені, що вже самі не свої, ви просто не впізнаєте себе. На цьому етапі для вашого психічного та фізичного здоров’я та/або добробуту дітей вам важливо зупинитись, вам потрібен відпочинок. Можливо, вам потрібна професійна допомога. Професійний психолог або психотерапевт може допомогти вам краще зрозуміти, як і чому ви в такому стані та надати вам рекомендації, що допоможуть «піднятись на ноги». Але вам необхідно попіклуватися про себе, по справжньому подбати про себе.

**Якщо ваш результат рівно 85 балів і більше:**

**Ви в стані батьківського вигорання**

Ви є одним з тих батьків, які саме в цей момент перебувають в стані батьківського вигорання. Ви виснажені, виснажені і ще раз виснажені. Іноді думка про новий день, що має розпочатися, здається для вас нестерпною. Ви все ще намагаєтесь триматися, але у вас залишається так мало енергії, що ви все частіше робите речі механічно: щоденні обов’язки - вечеря - сон. Забезпечуєте найнеобхідніше, слухаєте у пів-вуха...з думкою, що необхідно протриматися ще один день. Є гостре бажання, щоб діти сиділи тихо або вже були в ліжку. Часто з’являється почуття провини, що ви були недостатньо залучені або «не були присутні» по-справжньому у стосунках з дітьми. Маєте почуття провини за те, що злились або дратувались більше, ніж мали б. Ви усвідомлюєте, що ви вже не є тим татом або тією мамою, якими були раніше чи якими хотіли би бути. Ви бажаєте бути деінде, десь в іншому місці. В той же час ви відчуваєте себе винними, бо ви погана мама чи поганий батько. Ви виснажуєте себе почуттям провини, але все одно продовжуєте намагатись виконувати те, що необхідно. Часто ви на стільки стомлені, що вже самі не свої, ви просто не впізнаєте себе. Тепер вже все не так, як було раніше, все не так, як має бути. Ви не бачите жодного виходу. В такому стані, для вашого психічного та фізичного здоров’я та добробуту дітей, **критично необхідно**, щоб ви зробили зупинку і почали піклуватись про себе. Професійний психолог або психотерапевт може допомогти вам краще зрозуміти, як і чому ви в такому стані та надати вам рекомендації, що допоможуть вам відновитися, «піднятися на ноги». Вам терміново необхідно почати доглядати за собою, дійсно розпочати піклуватись про себе.
